# Extracellular vesicle-mimetic nanovesicles transport LncRNA-H19 as competing endogenous RNA for the treatment of diabetic wounds

**DOI:** 10.1080/10717544.2018.1425774

**Published:** 2018-01-15

**Authors:** Shi-Cong Tao, Bi-Yu Rui, Qi-Yang Wang, Ding Zhou, Yang Zhang, Shang-Chun Guo

**Affiliations:** a Department of Orthopedic Surgery, Shanghai Jiao Tong University Affiliated Sixth People’s Hospital, Shanghai, China;; b Department of Pharmacy, Shanghai Tenth People’s Hospital of Tongji University, Shanghai, China;; c Institute of Microsurgery on Extremities, Shanghai Jiao Tong University Affiliated Sixth People’s Hospital, Shanghai, China

**Keywords:** Long noncoding RNA, angiogenesis, diabetes, diabetic wound, nanovesicles, competing endogenous RNA

## Abstract

Diabetic wounds, one of the most enervating complications of diabetes mellitus, affect millions of people worldwide annually. Vascular insufficiency, caused by hyperglycemia, is one of the primary causes and categories of diabetic impaired wound healing. Recently, long noncoding RNA (LncRNA)-H19, which is significantly decreased in diabetes and may be crucial in triggering angiogenesis, has attracted increasing interest. The possible relationship between the decrease of LncRNA-H19 and the impairment of angiogenesis in diabetes could involve impairment of the insulin–phosphatidylinositol 3-kinase (PI3K)–Akt pathway via the interdiction of LncRNA-H19. Thus, a therapeutic strategy utilizing LncRNA-H19 delivery is feasible. In this study, we investigated the possibility of using high-yield extracellular vesicle-mimetic nanovesicles (EMNVs) as an effective nano-drug delivery system for LncRNA, and studied the function of EMNVs with a high content of LncRNA-H19 (^H19^EMNVs). The results, which were exciting, showed that ^H19^EMNVs had a strong ability to neutralize the regeneration-inhibiting effect of hyperglycemia, and could remarkably accelerate the healing processes of chronic wounds. Our results suggest that bioengineered EMNVs can serve as a powerful instrument to effectively deliver LncRNA and will be an extremely promising multifunctional drug delivery system in the immediate future.

## Introduction

Diabetic chronic wounds, one of the most enervating complications of diabetes mellitus (Tao et al., 2017a), are defined as barrier defects that have not proceeded through orderly and timely repair to regain structural and functional integrity (Bergan et al., 2006). Vascular insufficiency is one of the primary causes and categories of chronic non-healing cutaneous wounds, although systemic factors, including compromised nutritional or immunological status, advanced age, chronic mechanical stress, and other comorbidities, contribute to poor wound healing (Morton & Phillips, 2016). Poor wound healing affects millions of people worldwide each year and is the consequence of poorly-regulated elements of the healthy tissue repair response, especially angiogenesis (Ruttermann et al., 2013).

Competing endogenous RNAs (ceRNAs) are transcripts, including messenger RNAs (mRNAs), long non-coding RNAs (LncRNAs) and circular RNAs (circRNAs), which regulate each other by competitive binding to shared microRNAs (miRNAs) (Salmena et al., 2011). LncRNAs are recently-discovered non-coding forms of RNA which have been proven to participate in various biological processes and in several diseases (Zhang et al., 2016b). Accumulating evidence indicates that LncRNAs could contain miRNA-response elements (MRE), which could compete with mRNAs as ceRNAs (Cesana et al., 2011).

In the case of diabetic chronic wounds, hyperglycemia induces impairment of Akt activation (Vind et al., 2012) leading to defects in angiogenesis (Larger et al., 2004). Recent studies indicated that LncRNA-H19, which was first discovered from a genetic screen in 1984 (Pachnis et al., 1984), plays an important role in triggering angiogenesis (Jia et al., 2016). Interestingly, the expression level of LncRNA-H19 was significantly reduced in diabetes (Ding et al., 2012; Su et al., 2016). In addition, the H19/let-7 double-negative feedback loop led to impairment of the insulin–PI3K–Akt pathway (Gao et al., 2014), and the consequent inhibition of Akt activation caused failure of angiogenesis (Shiojima & Walsh, 2002).

Many types of cells generate phospholipid membrane extracellular vesicles including exosomes, which have emerged as a key form of intercellular communication (Kourembanas, 2015; Villasante et al., 2016; Tao et al., 2017b) by transferring various proteins, RNAs and even DNA between cells. Because they can protect encapsulated small RNAs from ribonucleases (RNases) in body fluid and have low antigenicity and toxicity (Burgess, 2014; Robbins & Morelli, 2014), extracellular vesicles are considered ideal carriers of nucleic acid drugs (Vader et al., 2016).

The present study explored the competence of extracellular vesicles as transporters of LncRNA to neutralize the regeneration-inhibiting effect of hyperglycemia and thus constitute a potential treatment strategy for diabetic wounds. To maximize the production of vesicles used for efficient LncRNA-H19 delivery, this study took advantage of high-yield extracellular vesicle-mimetic nanovesicles (EMNVs), whose production rate is over 100-fold greater than spontaneous release of extracellular vesicles from cells (Jang et al., 2013). HEK293 cells were considered to be an ideal source of extracellular vesicles and a proven protocol was available to produce extracellular vesicles as carriers for miRNA delivery (Ohno & Kuroda, 2016). However, there are few reports on the use of extracellular vesicles for LncRNA delivery.

In this study, for the first time, we investigated the function of EMNVs with a high content of LncRNA-H19 (^H19^EMNVs) and their possible molecular mechanisms in treating diabetic wounds. The results indicated that EMNVs transporting LncRNA as ceRNA will lay the foundation for new strategies of regenerative medicine.

## Methods

### Cells and cell culture

Human dermal microvascular endothelial cells (HMEC-1) and human embryonic kidney cells 293 (HEK293) were obtained from the Cell Bank of the Chinese Academy of Sciences (Shanghai, China). HMEC-1 were cultured in MCDB131 medium (Gibco, Thermo Fisher Scientific Inc., Waltham, MA) supplemented with 10% fetal bovine serum (FBS, Gibco), 2 mM l-glutamine (Gibco), 10 ng/mL epidermal growth factor (EGF, Sigma-Aldrich, St. Louis, MO) and 1 μg/mL hydrocortisone (Sigma-Aldrich). HEK293 cells were cultured in Dulbecco’s modified Eagle medium (DMEM; Gibco) supplemented with 10% FBS (Gibco), 100 U/mL penicillin and 100 μg/mL streptomycin sulfate (Gibco). Both cell types were cultured in a humidified atmosphere (5% CO_2_, 37 °C), with medium changes twice a week. BMS-754807 (referred to as RI in this article), a selective small molecule receptor inhibitor of IGF-1R and InsR (Carboni et al., 2009; Wittman et al., 2009; Kolb et al., 2011), was obtained from Selleck (Houston, TX) and used at a concentration of 1 μM for *in vitro* assays.

### H19 overexpression and silencing

The H19-overexpressing (H19-OE) lentiviral vector was purchased from Obio Technology (Shanghai, China). Cell transfection was performed following the protocol provided by the supplier. Briefly, cells were incubated in lentiviral supernatant with 5 μg/mL polybrene for 24 h. Forty-eight hours after infection, cells were selected with 2.5 μg/mL puromycin (Sigma-Aldrich) in culture medium.

LncRNA-H19 Smart Silencer (H19-SS) and negative control (NC) were purchased from RiboBio (Guangzhou, China) and transfected according to the manufacturer’s protocol.

### Preparation of extracellular vesicle-mimetic nanovesicles (EMNVs)

The preparation of EMNVs was based on the methods described by Jang et al. (2013) HEK293 cells were first transfected with an H19-OE lentiviral vector or empty vector and selected using puromycin, after which they were considered as the raw materials of EMNVs. After harvesting with TrypLE™ Express (Gibco) and washing with Dulbecco's phosphate-buffered saline (DPBS, Corning, Corning, NY), cells were diluted to 5 × 10^6^ cells/mL with DPBS and extruded serially through 10 μm, 5 μm and 1 μm polycarbonate filters (Whatman, Maidstone, UK) with an extruder kit (Avanti Polar Lipids, Alabaster, AL) three times. The extrusion product was finally extruded through a 0.2 μm polycarbonate filter. For further isolation and purification of EMNVs, the final extrusion solution was transferred to a 15 mL Amicon Ultra-15 Centrifugal Filter Unit (Merck–Millipore) and centrifuged at 4000 × *g* for 30 min. After washing with DPBS three times, the ultrafiltered liquid was laid on the top of a 30% sucrose/D_2_O cushion in a sterile Ultra-Clear™ tube (Beckman Coulter, Brea, CA) and centrifuged at 120,000 × *g* for 90 min. All centrifugation steps were performed at 4 °C. After obtaining the EMNVs, they were stored at −80 °C or used in subsequent experiments. The protein concentration of the EMNVs was measured using the Pierce™ BCA Protein Assay Kit (Thermo Fisher Scientific Inc.), which is widely used in the quantification of extracellular vesicles (El-Andaloussi et al., 2012; Perez-Gonzalez et al., 2012).

A dynamic light scattering (DLS) system, known as Nanosizer™ technology (Malvern Instruments, Malvern, UK) was used to examine the size distribution of EMNVs, and a Hitachi H-7650 transmission electron microscope (TEM, Japan) was used to observe the morphologies of EMNVs. Western blotting was used to examine the specific exosome surface markers, CD9, CD63 and CD81, which could also be expressed in EMNVs.

### EMNV uptake by endothelial cells

HEK293 cells were labeled with Vybrant™ DiL dye (Molecular Probes, Thermo Fisher Scientific) following the manufacturer’s instructions. Briefly, cells were digested and resuspended in 1 mL of serum-free DMEM, then 5 µL of DiL dye solution was added to the cell suspension and incubated with cells at 37 °C for 15 min. The labeled cell suspension was centrifuged at 300 × *g* for 5 min and the supernatant was discarded. The cells were resuspended with DPBS and centrifuged at 300 × *g* for 5 min, three times, before being cultured for 48 h. EMNVs were harvested following the aforementioned steps and incubated with HMEC-1 at 37 °C for 2 h. After treatment with EMNVs, cells were fixed with 4% paraformaldehyde for 15 min, stained with DAPI for 5 min and then stained with Alexa Fluor^®^ 488 Phalloidin (Thermo Fisher Scientific) for 25 min. Cells were then analyzed under a fluorescence microscope (Leica DMI6000B, Leica Microsystems Ltd, Wetzlar, Germany).

### Preparation of EMNV-loaded sodium alginate hydrogel

Sodium alginate (SA) was purchased from Sigma-Aldrich, and sodium alginate hydrogel (SAH) was prepared according to the procedure of Blandino et al. (1999). Briefly, to prepare EMNV-loaded hydrogel, a weighed quantity of SA dry powder (1.25% w/v) was mixed in sterile distilled water and agitated for 1 h to dissolve, before ^H19^EMNVs, ^293^EMNVs or the same volume of DPBS (1% v/v) was added to the solution under gentle agitation. The resultant mixtures were then poured into a 24-well plate filled with calcium chloride solution (5% w/v), then kept at room temperature for 48 h to gel. Next, the redundant calcium chloride solution was discarded from the well and the EMNV-loaded SAH samples were air-dried at room temperature. The resultant EMNV-loaded SAH formed discs of 1.8 cm diameter and almost 2.0 mm thickness.

### Cell proliferation assay using flow cytometry

The effect of ^293^EMNVs and ^H19^EMNVs on the proliferation of HMEC-1 was measured using an EdU Cell Proliferation Kit-488 with flow cytometry (EdU-488, RiboBio, Guangzhou, China) following the manufacturer’s protocol. In brief, HMEC-1, at an initial density of 2 × 10^4^ cells/well, were seeded into 48-well plates and cultured in normal (5.56 mM glucose according to the ingredient list provided by the manufacturer) or high glucose (HG, 25 mM glucose) medium containing ^293^EMNVs (50 μg/mL) or ^H19^EMNVs (50 μg/mL) for 24 h as pretreatment. Then 150 μL of specific culture medium, described above, mixed with 0.15 μL of EdU was added into each well and incubated at 37 °C. After incubation for 2 h, cells were digested, centrifuged, washed with DPBS twice and fixed with 4% paraformaldehyde for 15 min. After neutralizing with 2 mg/mL glycine, cells were washed with DPBS twice, permeabilized with 0.5% Triton X-100 for 10 min and washed with DPBS. Next, cells were resuspended using Apollo staining solution (RiboBio), and incubated for 10 min. Cells were washed three times using 0.5% TritonX-100 and resuspended with DPBS. Finally, cells were analyzed using an easyCyte™ flow cytometer (Merck-Millipore, Darmstadt, Germany).

### Cell proliferation assay using cell counting kit-8

We used the Cell Counting Kit-8 (CCK-8; Dojindo Molecular Technologies, Inc., Kumamoto, Japan) to observe the proliferative ability after the different treatments. The detailed procedures to perform this assay were as described previously (Guo et al., 2017).

### Cell migration

The effect of ^293^EMNVs or ^H19^EMNVs on HMEC-1 migration was carefully analyzed using a transwell assay. Briefly, 2 × 10^5^ HMEC-1 cells, transfected with empty vector, H19-OE, negative control (NC) or H19-SS, were plated into the upper chamber of a 24-well transwell plate (Corning, pore size = 8 μm). Then 700 μL/well of normal medium or HG medium containing ^293^EMNVs (50 μg/mL), ^H19^EMVNs (50 μg/mL) or ^H19^EMNVs (50 μg/mL) together with RI (1 μM) were added to the lower chamber. After incubation for 12 h, cells that migrated to the lower surface of the filter membrane were stained with 0.5% crystal violet for 15 min and washed three times with DPBS. Cells on the upper surface of the filter membranes were removed with a cotton swab. Migratory activity was evaluated by observing and counting the stained cells under an optical microscope.

### Vessel-like formation assays

To investigate the capillary-like construction formation of HMEC-1 cells, briefly, 150 μL of cold ECM gel (Sigma-Aldrich) per well was transferred with a pre-cooled tip to a 48-well plate. After gelling for 15 min at 37 °C, the ECM gel was overlaid with 150 μL of a suspension of HMEC-1 cells (2 × 10^4^ cells/well), which were transfected with empty vector, H19-OE, NC or H19-SS, and pretreated with normal medium or HG medium containing ^293^EMNVs (50 μg/mL), ^H19^EMNVs (50 μg/mL) or ^H19^EMNVs (50 μg/mL) together with RI (1 μM). Tube formation capacity was assessed by observing the polygonal structures formed 12 h after seeding the cells onto the ECM gel, using a light microscope. Two well-recognized statistical methods – percentage of tube numbers relative to control (Amin et al., 2010) and percentage of branch points relative to control (Eubank et al., 2004) – were used in the statistical analysis in this study.

### Reverse transcription-polymerase chain reaction(RT-PCR)

Total RNA was extracted from cells with TRIzol Reagent and from EMNVs with a Total Exosome RNA & Protein Isolation Kit (both from Invitrogen, Carlsbad, CA) referring to the guidebook provided. For mRNA and LncRNA, cDNA was synthesized using a TransScript All-in-One First-Strand cDNA Synthesis SuperMix (Transgen Biotech, Beijing, China) and PCR was performed using 2 × Taq PCR MasterMix (Solarbio, Beijing, China) according to the steps described in the handbooks.

Primers used in the present study are shown in Table 1.

**Table 1.** Primers used for PCR.

Gene name	Forward primer	Reverse primer
Beta-actin	AAGGTGACAGCAGTCGGTT	TGTGTGGACTTGGGAGAGG
H19	AAAGCCTCCACGACTCTGTT	GCTCACACTCACGCACACTC

Further, quantitative PCR (qPCR) was performed following the procedures described previously (Tao et al., 2017d) for precise quantification.

### Polyacrylamide gel electrophoresis (PAGE) of the PCR products

The PAGE procedure for the PCR products followed the manufacturer’s instructions in the guidebooks provided by Solarbio. Briefly, SYBR Green I (10,000×, Solarbio) was diluted into SYBR Green I (10×) with sterilized deionized water (Solarbio). SYBR Green I (10×) and 6 × DNA Loading Buffer (Solarbio) were then mixed at a ratio of 1:1. Next, the PCR products and the above mixture were combined at a ratio of 2:1. The mixed samples were added into the wells of a preformed PAGE gel (Solarbio) and electrophoresed at 150 V for 40 min. After electrophoresis, the gel was examined using the Gel Doc™ XR + system (Bio-Rad, Hercules, CA).

### Western blot analysis

Protein lysates were prepared from cells using RIPA buffer (BioTNT, Shanghai, China) supplemented with a cocktail of protease and phosphatase inhibitors (BioTNT). Protein lysates of EMNVs were prepared using a Total Exosome RNA & Protein Isolation Kit. Lysates were diluted at a ratio of 1:5 with protein loading buffer (6×, Transgen Biotech) and heated at 95 °C for 5 min. Protein extracts were separated on a 10% FastPAGE Plus premixed gel (New Cell & Molecular Biotech, Suzhou, China) at 120 V for 1 h and blotted onto a polyvinylidene difluoride (PVDF) membrane (Merck-Millipore) for 90 min at 200 mA. The membranes were then blocked for 120 min with 5% nonfat milk in TBST (Tris-buffered saline, 10 mM Tris-HCl pH 7.5, 150 mM NaCl, 0.1% Tween-20). Subsequently, the membranes were incubated with primary antibodies at 4 °C overnight, followed by incubation with horseradish peroxidase (HRP)-labeled secondary antibodies (Cell Signaling Technology, Danvers, MA) at 37 °C for 1 h. The immunoreactive bands were visualized using enhanced chemiluminescence reagent (Thermo Fisher Scientific) and imaged using an Image Quant LAS 4000 mini bio-molecular imager (GE Healthcare, Uppsala, Sweden).

### Antibodies for Western blotting

The primary antibodies used were anti-CD9, anti-CD63, anti-CD81 (System Biosciences, Palo Alto, CA); anti-insulin receptor β (InsR), anti-IGF-I receptor β (IGF-1R), anti-AKT and phosphorylated AKT (p-AKT) and anti-ACTB (Cell Signaling Technology).

### Preparation of the diabetic rat model and experimental groups

The ability of ^H19^EMNVs to promote healing of chronic skin wounds was evaluated in a diabetic rat model, which is a widely-recognized model used to evaluate therapeutic effects in diabetes-induced impaired tissue regeneration (Grotendorst et al., 1985; Iwakura et al., 2001; Kuo et al., 2009; Lai et al., 2014). Streptozotocin (STZ) was prepared in a 0.1 M phosphate–citrate buffer (pH 4.5). Sprague–Dawley (SD) rats were examined on the third day following STZ administration to confirm initiation of the induction of diabetes. Tail vein blood samples were taken, and blood glucose levels >250 mg/dL (13.9 mmol/L), measured using a glucometer (Roche, Basel, Switzerland), were regarded as indicating diabetes in this study. The rats’ blood glucose levels and weight changes were measured every day for two weeks after diabetes induction. The study was conducted once the induction of diabetes was confirmed in the rat model.

Anesthesia was administered by intraperitoneal (i.p.) injection of 3% pentobarbital sodium solution (Sigma-Aldrich) at a dose of 1.0 mL/kg before operation. Defect areas were marked precisely with a round-shaped seal and standardized full-thickness skin wounds (diameter = 1.8 cm) were prepared by resecting the dorsum under aseptic conditions.

Forty-five rats were randomly divided into four groups (*n* = 9/group) as follows: (1) Control, untreated skin defects; (2) SAH, defects treated with SAH; (3) SAH-^293^EMNVs, defects treated with ^293^EMNVs-loaded SAH; (4) SAH-^H19^EMNVs, defects treated with ^H19^EMNVs incorporated into SAH; and (5) SAH-^H19^EMNVs + RI, defects treated with ^H19^EMNVs incorporated into SAH, while the rats were treated orally by RI (2.5 mg/kg, daily). After the wound beds were treated by one of the five different methods, the rats received a pressure dressing to protect the wound. At 3, 7 and 14 d after operation, three rats from each group were randomly and blindly chosen and sacrificed. After blood vessel perfusion (described later), skin specimens were harvested. Skin samples were first analyzed by measuring any reduction in wound size, then micro-computed tomography (micro-CT) was used for blood vessel analysis. Finally, every skin sample was analyzed by histological and IF methods.

### Measurement of wound closure

After operation, the wounds were photographed at 0, 3, 7 and 14 d with a Nikon D810 digital camera (Nikon, Japan) and Sigma 50 mm F1.4 Art standard lens (Sigma-Photo, Kanagawa, Japan). The margin of each wound was traced and the wound area was measured using ImageJ (National Institutes of Health, Bethesda, MD).

The size of wound closure was calculated using the formula below:

$$\text{Percentage wound size reduction }=\;[({\text{W}}_{0}-{\text{W}}_{\text{t}})/{\text{W}}_{0}]\times \text{1}00,$$

where W_0_ is the initial wound area (t = 0) and W_t_ is the wound area at each time — point.

### Microfil wound blood vessel perfusion with micro-CT

Microfil wound blood vessel perfusion is a widely-used method to evaluate blood vessel regeneration (Zhao et al., 2015). The rats were perfused with Microfil (Microfil MV-122; Flow Tech, Carver, MA) before they were euthanized. Briefly, after anesthesia, the hair of the chest was shaved and the rib cage was opened with a pair of scissors. Then, 100 mL of heparinized saline and 20 mL of Microfil were continuously perfused at a rate of 2 mL/min. The rats were laid flat at 4 °C in the refrigerator overnight to ensure complete polymerization before the tissue containing the wound bed and surrounding healthy skin was collected and fixed using paraformaldehyde. Then the wounds were scanned by micro-CT (Skyscan 1176 high-resolution *in vivo* micro-CT scanner; Bruker, Billerica, MA) at a resolution of 9 μm to observe blood vessels. Three-dimensional images were reconstructed with the CTVol program (Bruker). The area and number of blood vessels in each defect were also determined with the software ImageJ.

### Histologic and immunofluorescence (IF) analysis

The wound samples were fixed in paraformaldehyde, dehydrated through a graded series of ethanol and embedded in paraffin. Sections (∼5 μm thick) were stained with Hematoxylin and Eosin (HE) and Masson's trichrome and examined under an optical microscope. The renascent skin length was assessed using a method described previously (Nakamura et al., 2013).

IF staining of thin sections was performed to study angiogenesis during the wound-healing process. IF staining of CD31 (1:50, Abcam), a marker of vascular endothelial cells, and alpha-smooth muscle actin (α-SMA, 1:200, Abcam), a marker of vascular smooth muscle cells, were used to estimate the extent of the regenerated mature vessels. In addition, anti-p-Akt and anti-cytokeratin 14 (K14; 1:100, Abcam) antibodies were used as primary antibodies for IF staining.

The sections were rehydrated, blocked with 1.5% goat serum (Merck-Millipore), and incubated in the primary antibody overnight at 4 °C. After treating the specimens with Alexa Fluor 488- or Cy3-conjugated secondary antibodies, DAPI (Sigma-Aldrich) was used to stain the nuclei. The images were examined with an LSM-880 confocal-microscope (Carl Zeiss, Oberkochen, Germany). For each section, three high-power fields were randomly captured and the regenerative mature vessels were blindly evaluated by two different pathologists.

### Statistical analysis

All data are presented as means ± standard deviation (SD). Student's *t* test was used to determine the significance when only two value sets were compared and one-way analysis of variance (ANOVA) was used when data involved three or more groups. Statistical analysis was carried out and plotted using GraphPad Prism (GraphPad Software, Inc.). *p* values < .05 were considered statistically significant.

### Study approval

The use of animals in this study followed the Interdisciplinary Principles and Guidelines for the Use of Animals in Research, Testing, and Education. All animal experiments complied with the ARRIVE guidelines. All experimental and animal care procedures were approved by the Animal Research Ethics Committee of Shanghai Sixth People’s Hospital and were performed in accordance with the National Institutes of Health Guidelines for the Care and Use of Laboratory Animals.

## Results

### Preparation and characterization of EMNVs

**Figure 1.**
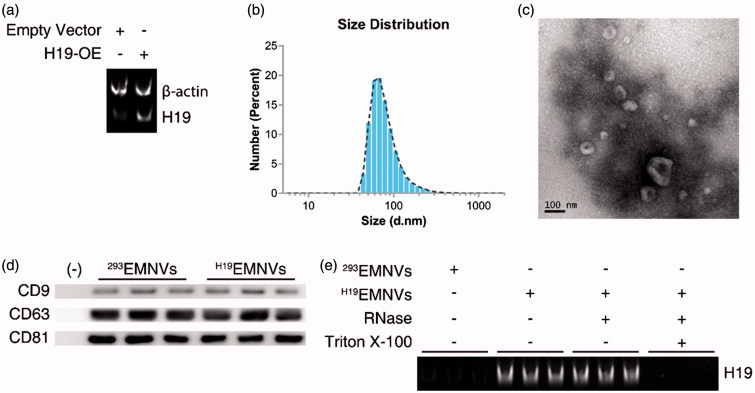
Preparation and characterization of EMNVs. (a) PAGE analysis immediately after RT-PCR in HEK-293 cells transfected with empty vector or H19-OE. (b) The size distribution of EMNVs directly tracked using a DLS system. (c) Representative TEM images of EMNVs; scale bar, 100 nm. (d) Western blot analysis of CD9, CD63 and CD81. (e) PAGE analysis immediately after RT-PCR (initial total RNA content of each sample set as 100 ng) in ^293^EMNVs and ^H19^EMNVs treated with RNase (2 mg/mL) alone or combined with Triton X-100 (0.1%) for 15 min.

HEK293 (human embryonic kidney 293) cells were transfected with an H19-overexpressing (H19-OE) lentiviral vector and purified using puromycin, to establish a stable H19-high-expressing HEK293 cell line (H19-293). The H19 expression levels were analyzed using reverse transcription–polymerase chain reaction (RT-PCR) and polyacrylamide gel electrophoresis (PAGE) to ascertain the efficiency of overexpression (Figure 1(a)). The EMNVs derived from HEK-293 cells transfected by empty vector (^293^EMNVs) and stable H19-high-expressing HEK293 cells (^H19^EMNVs) were prepared and purified following the procedure described in the “Methods” section.

The size distribution of ^H19^EMNVs was directly tracked using Nanosizer, a dynamic light scattering (DLS) system, and ^H19^EMNVs were found to have a mean diameter of 82.15 ± 40.60 nm (Figure 1(b)). Transmission electron microscopy (TEM) examination of the ^H19^EMNVs showed that the vast majority of these nanoparticles exhibited a cup- or sphere-shaped morphology, extremely similar to exosomes (Figure 1(c)), indicating the presence of EMNVs. Western blotting showed the presence of exosome markers, such as CD9, CD63, and CD81 (Figure 1(d)), which further confirmed the similarities between extracellular vesicles and EMNVs. All these data suggested that these nanoparticles were EMNVs. The content of H19 was much higher in ^H19^EMNVs than in ^293^EMNVs, when analyzed by RT-PCR and PAGE (Figure 1(e)). In consideration of the relative stability of the LncRNA to exist in their free form in the culture supernatant, RNase and Triton X-100 were used to confirm the location of the over-expressed LncRNA-H19, as a generally accepted method (Qu et al., 2016). The levels of LncRNA-H19 remained unchanged after RNase treatment but dramatically reduced after simultaneous treatment with RNase and Triton X-100, indicating that over-expressed LncRNA-H19 was mainly enveloped within EMNVs (Figure 1(e)).

### Internalization of EMNVs by endothelial cells

**Figure 2.**
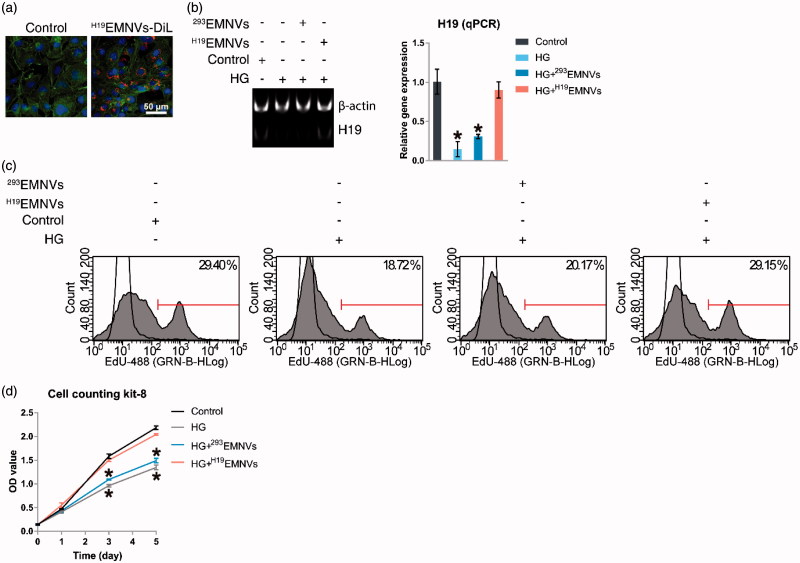
^H19^EMNVs as transporters of LncRNA-H19. (a) Uptake of DiL-labeled ^H19^EMNVs (^H19^EMNVs-DiL) by HMEC-1. Scale bar, 50 μm. (b) PAGE analysis immediately after RT-PCR in HMEC-1 cultured in normal medium or in HG medium with or without ^H19^EMNVs or ^293^EMNVs, along with the results of real-time qPCR. *p < .05 compared with control. (c) Proliferation of HMEC-1 analyzed by flow cytometry (FCM) using an EdU kit after culture in normal medium or HG medium with or without ^H19^EMNVs or ^293^EMNVs. (d) Proliferation of HMEC-1 detected using a CCK-8 kit on day 0, 1, 3 and 5 in normal medium or HG medium with or without ^H19^EMNVs or ^293^EMNVs. *p < .05 compared with control.

Fluorescent lipophilic dye is a recognized and convenient tool used to trace exosomes (Wang et al., 2012; Boelens et al., 2014; Zhang et al., 2016a), one type of extracellular vesicles, so we considered that since EMNVs are similar to exosomes it would be suitable for tracing EMNVs. To investigate whether ^H19^EMNVs could be taken up by human dermal microvascular endothelial cells (HMEC-1), H19-293 cells were labeled using red fluorescent lipophilic dye (Vybrant DiL) before extraction of ^H19^EMNVs. After isolation, DiL-labeled ^H19^EMNVs (^H19^EMNVs-DiL) were incubated with HMEC-1, and the ^H19^EMNVs-DiL could be observed in the perinuclear region of the HMEC-1 by fluorescence microscopy (Figure 2(a)), demonstrating internalization by HMEC-1.

### ^H19^EMNVs rescued the hyperglycemia-induced reduction of LncRNA-H19

Based on previous studies (Dunn et al., 2014; Yoon et al., 2014), we defined 25 mM glucose as high glucose (HG), and defined 5.56 mM glucose, as described in the ingredient list of conventional medium provided by the manufacturer, as normal glucose. HMEC-1 were cultured in normal medium or HG medium with or without ^H19^EMNVs or ^293^EMNVs. The H19 level was found to be markedly inhibited by HG medium and rescued by ^H19^EMNVs, while ^293^EMNVs had no obvious effect (Figure 2(b)). This result indicated that H19 could be transported into HMEC-1 by ^H19^EMNVs to compensate for the hyperglycemia-induced deficiency of H19. It is important to add that the promotion of cell proliferation, which is well recognized as one of the major functions of LncRNA-H19 (Ghazal et al., 2015), showed the same trend (Figure 2(c,d)).

### Hyperglycemia-induced impairment of Akt activation by interdicting LncRNA-H19

**Figure 3.**
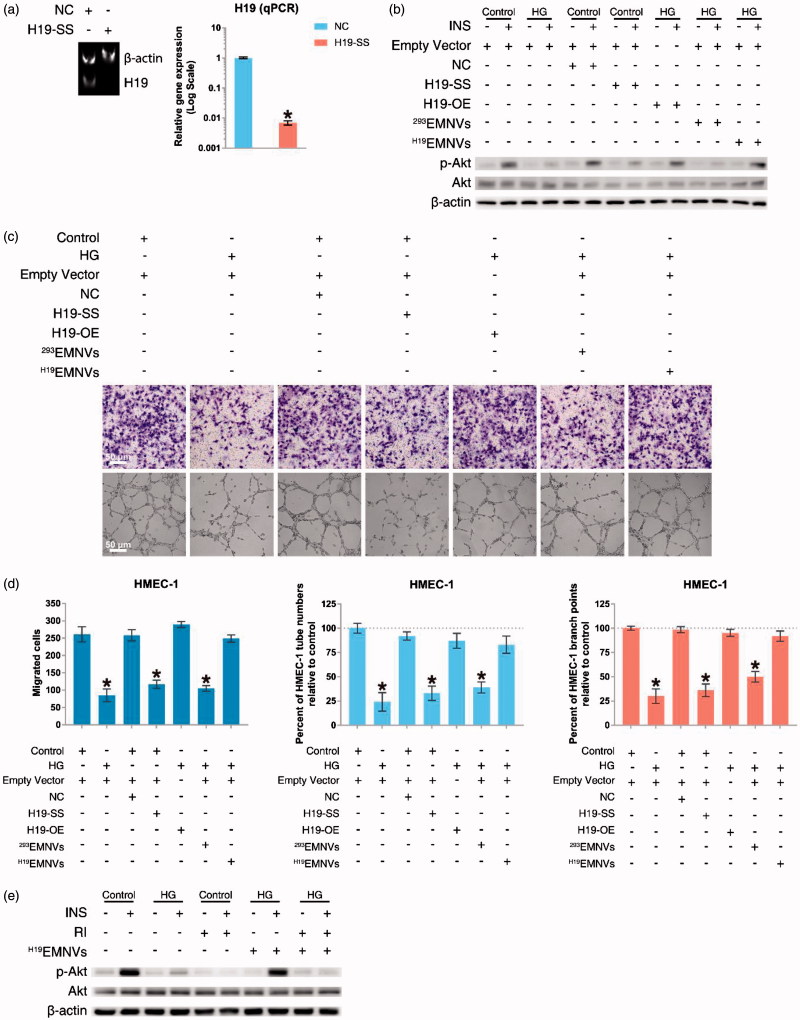
^H19^EMNVs rescued the hyperglycemia-induced impairment of angiogenesis by sustaining the vitality of Akt activation through regulation of the LncRNA-H19. (a) PAGE analysis immediately after RT-PCR in HMEC-1 transfected with NC or H19-SS, along with the results of real-time qPCR. *p < .05 compared with NC. (b) Western blot analysis of the phosphorylation level of Akt. (c) Representative photomicrographs of transwell assays; scale bar, 50 μm, and representative photomicrographs of tubule formation; scale bar, 50 μm. (d) Statistical results of (c) in migrated cells, percentage of HMEC-1 tube numbers relative to control and percentage of HMEC-1 branch points relative to control. *p < .05 compared with control. (e) Western blot analysis of the phosphorylation level of Akt.

To verify the efficiency of Ribo™ LncRNA Smart Silencer for LncRNA-H19 (H19-SS), HMEC-1 were transfected with negative control (NC) or H19-SS. The result showed that the expression level of LncRNA-H19 could be successfully inhibited by H19-SS (Figure 3(a)). After transfection with empty vector, H19-OE, NC or H19-SS, HMEC-1 were cultured in normal medium or HG medium with or without ^H19^EMNVs or ^293^EMNVs for 48 h, and then stimulated by insulin (INS) after serum starvation. The result demonstrated that hyperglycemia could induce impairment of Akt activation and confirmed that the underlying mechanism would be the interdiction of LncRNA-H19, while ^H19^EMNVs could restore the vitality of Akt (Figure 3(b)).

The well-recognized methods used to evaluate angiogenic function are migration assays and tubule formation assays, both of which have been used to observe the high-glucose-induced impairment of angiogenic function (Dunn et al., 2014). Our results showed that the capability of migration and angiogenesis corresponded with Akt activity (Figure 3(c,d)). These results suggested that the interdiction of LncRNA-H19 is highly related to the high-glucose-induced impairment of angiogenesis via the devitalization of Akt.

Furthermore, we confirmed the loss of insulin receptor (InsR) and insulin-like growth factor-1 receptor (IGF-1R) induced by the loss of LncRNA-H19 (Figure S1a), which has been described previously (Gao et al., 2014; Ghazal et al., 2015). Considering that these two receptors could be the ‘hub’ in the connection between LncRNA-H19 and the impairment of Akt activation, we obtained a selective small molecule receptor inhibitor of IGF-1R and InsR, BMS-754807 (hereafter shortened to ‘RI’) (Carboni et al., 2009; Wittman et al., 2009; Kolb et al., 2011), and confirmed that RI could block the effect of ^H19^EMNVs in rescuing Akt activation (Figure 3(e)) as well as angiogenic ability (Figure S1b and c).

### Evaluation of wound healing following ^H19^EMNV treatment *in vivo*

**Figure 4.**
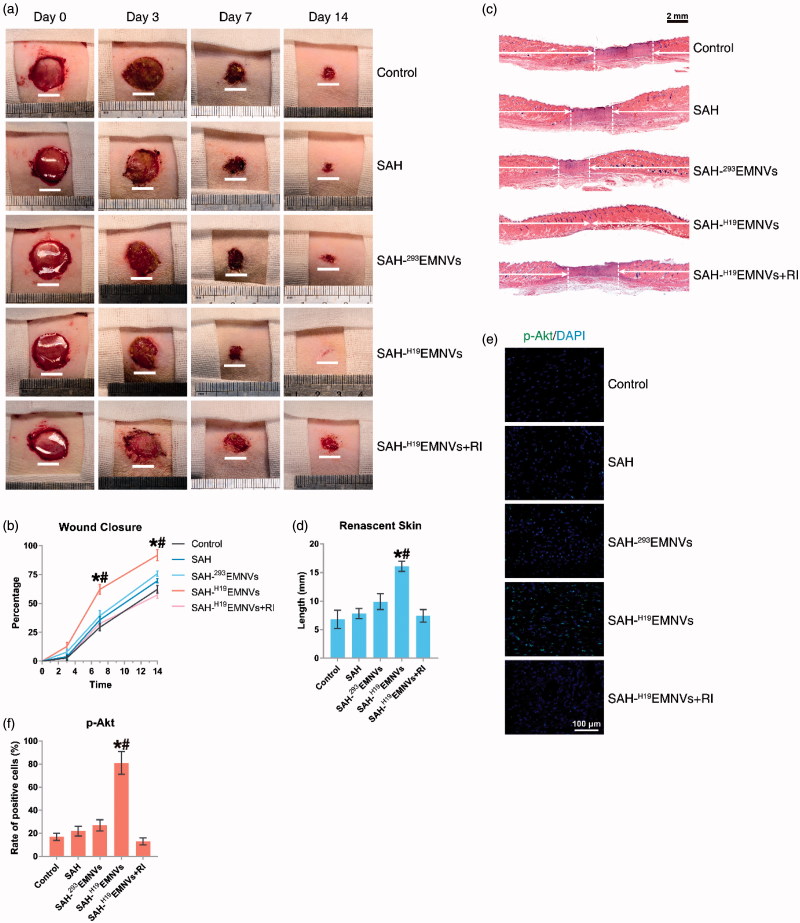
^H19^EMNVs promoted healing of diabetic wounds. (a) Representative images of full-thickness skin defects in a diabetic rat model, left untreated (control) or treated with SAH, SAH-^293^EMNVs, SAH-^H19^EMNVs or SAH-^H19^EMNVs together with RI (BMS-754807), at 0, 3, 7 and 14 days after operation. Scale bar: 10 mm. (b) Percentage wound closure of untreated defects and defects treated with SAH, SAH-^293^EMNVs, SAH-^H19^EMNVs or SAH-^H19^EMNVs together with RI (BMS-754807) at 3, 7 and 14 days after surgery. *p < .05 compared with control. #*p* < .05 compared between SAH-^H19^EMNVs and SAH-^293^EMNVs. (c) Transmitted light images of HE-stained sections of the untreated defects (control) and the defects treated with SAH, SAH-^293^EMNVs, SAH-^H19^EMNVs or SAH-^H19^EMNVs together with RI (BMS-754807) at 14 days after operation (scale bar = 2 mm). The total width of the image represents the initial defect size (1.8 cm) while the white arrows indicate the length of full thickness wound healing. (d) Total length of full thickness wound healing (renascent skin) in the skin defects left untreated (control), treated with SAH, SAH-^293^EMNVs, SAH-^H19^EMNVs or SAH-^H19^EMNVs together with RI (BMS-754807) at 14 days after operation. *p < .05 compared with control. #*p* < .05 compared between SAH-^H19^EMNVs and SAH-^293^EMNVs. (e) Immunofluorescence images of p-Akt counterstained with DAPI; scale bar, 100 μm. (f) Rate of positive cells (%) in (e). *p < .05 compared with control. #*p* < .05 compared between SAH-^H19^EMNVs and SAH-^293^EMNVs.

No adverse effects were observed at any time-point during the *in vivo* experimental procedure. Digital photographs showed the progress of closure of the untreated wounds and those treated with SAH-^H19^EMNVs with or without RI, SAH-^293^EMNVs and SAH at 0, 3, 7 and 14 days (Figure 4(a)). While the wounds in all five groups contracted over time, the reduction in wound size in the SAH-^H19^EMNVs group was faster than in the untreated group at all time-points evaluated, but the SAH, SAH-^293^EMNVs or SAH-^H19^EMNVs + RI groups did not show such significant effects. In particular, the wounds treated with SAH-^H19^EMNVs had almost closed by day 14.

Quantification of the wound closure confirmed that the wounds treated with SAH-^H19^EMNVs closed significantly faster than the untreated wounds at day 7 and 14, but the other groups did not show such a significant improvement (Figure 4(b)).

### Histological analyses of wound healing

Light micrographs of HE-stained sections revealed the persistence of full thickness wounds in the untreated group and those treated with SAH-^H19^EMNVs with or without RI, SAH-^293^EMNVs, and SAH at 14 days (Figure 4(c)). The width of the photograph indicates the total initial wound size (1.8 cm). The wounds treated with SAH-^H19^EMNVs appeared to show significantly better full thickness skin regeneration than the untreated wounds and the length of the renascent skin of the wounds treated with the SAH-^H19^EMNVs was significantly longer than that of the untreated wounds at day 14, while the other groups did not show such significant effects (Figure 4(c,d)). Furthermore, the phosphorylation level of Akt in the SAH-^H19^EMNVs group was much higher than in the untreated group but the other groups did not show such a significant effect (Figure 4(e,f)).

**Figure 5.**
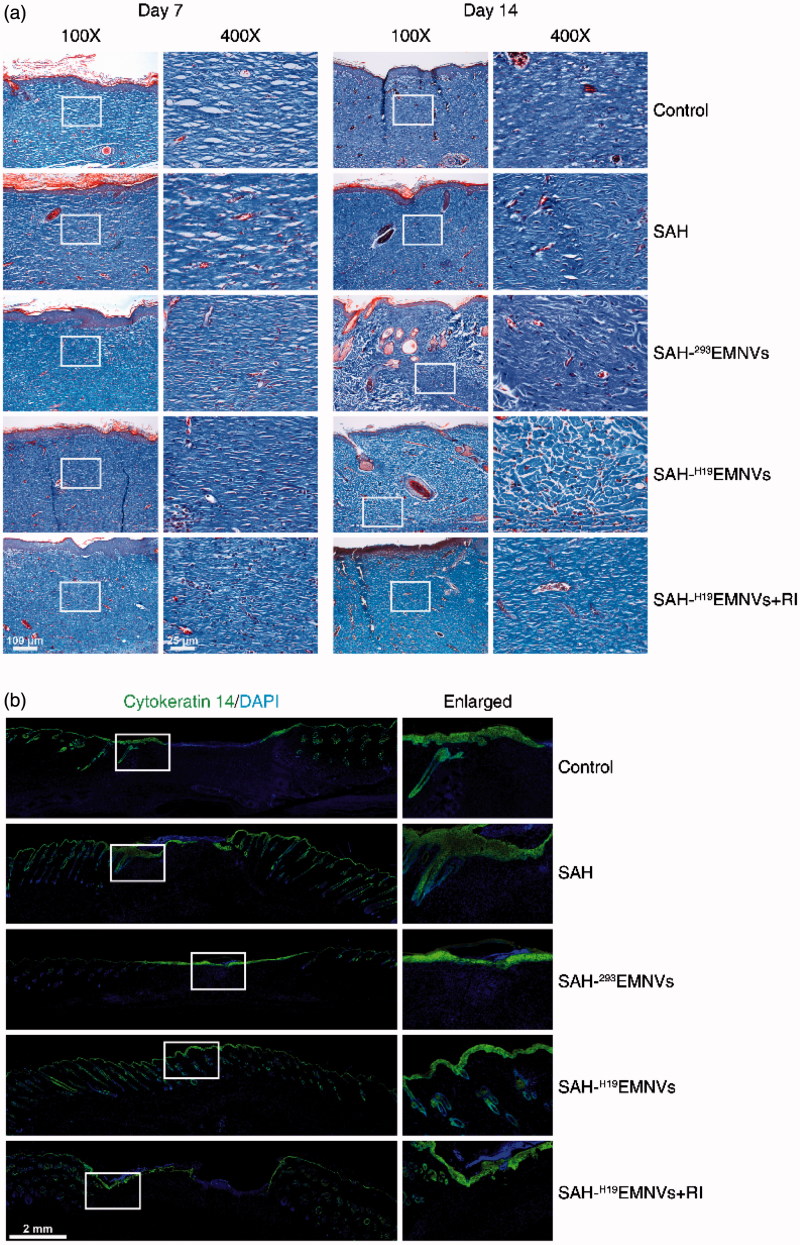
The deposition and remodeling of collagen, and the regeneration of epithelium. (a) Transmitted light images of Masson's trichrome-stained sections of the untreated defects (control) and the defects treated with SAH, SAH-^293^EMNVs, SAH-^H19^EMNVs or SAH-^H19^EMNVs together with RI (BMS-754807) at 7 and 14 days after operation, showing collagen deposition. Scale bar, 100 μm (100×), 25 μm (400×). (b) Immunofluorescence images of cytokeratin 14 (K14) counterstained with DAPI; scale bar, 2 mm.

Histological analysis of Masson's trichrome-stained sections revealed differences in the repair efficiency of the wounds among all five treatment groups at different time-points (Figure 5(a)). When the appearance of the wound was compared to that of untreated wounds, the depth, deposition of large amounts of collagen and the presence of large wavy collagen fibers were all observed to be improved in the defects treated with SAH-^H19^EMNVs. Photographs of the wounds treated with SAH-^H19^EMNVs revealed the presence of larger amounts of collagen fibers that were arranged in a well-organized matrix similar to normal skin, indicating the enhancing effect of the SAH-^H19^EMNVs on collagen remodeling. Generally, there were more constructions that resembled hair follicles and sebaceous glands in the defects treated with SAH-^H19^EMNVs than in any of the other groups.

The results of cytokeratin 14 (K14) staining, shown in Figure 5(b), are very intriguing. SAH could, to some extent, help the regeneration of epithelium, possibly due to its anti-inflammatory action (Jeong et al., 2006) and regulatory effects on mammary epithelial cells (Chaudhuri et al., 2014). To a certain extent, the effects of ^293^EMNVs on the regeneration of epithelium might be caused by some original factors carried by EMNVs. However, it is quite obvious that, without the addition of LncRNA-H19, they do not have a sufficiently powerful angiogenic effect, and ultimately lead to the absence of full thickness wound healing, appearing as the impaired regeneration of dermis, vividly described as ‘shiny on the “outside”, broken on the “inside”’. Furthermore, under the much worse angiogenic conditions caused by RI, the regeneration of epithelium was also inhibited to some extent.

### Conditions of blood supply

**Figure 6.**
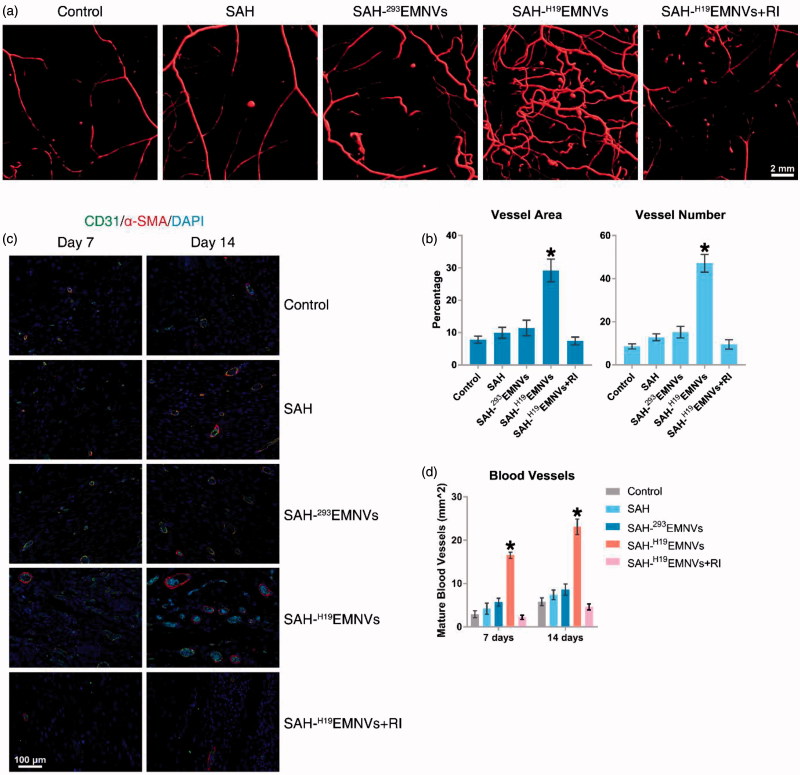
^H19^EMNVs promoted angiogenesis in diabetic wounds. (a) Micro-CT evaluation of blood supply in full-thickness skin defects left untreated (control) or treated with SAH, SAH-^293^EMNVs, SAH-^H19^EMNVs or SAH-^H19^EMNVs together with RI (BMS-754807) at 14 days after surgery; scale bar, 2 mm. (b) Morphometric analysis of the new blood vessel area and the number of blood vessels. *p < .05 compared with control. (c) IF staining of CD31 and α-SMA. Endothelial cells (CD31), smooth muscle cells (α-SMA) and cell nuclei are stained. CD31 and ?-SMA co-staining indicates mature blood vessels. Scale bar: 100 μm. (d) Number of regenerated mature blood vessels in the untreated defects (control) and the defects treated with SAH, SAH-^293^EMNVs, SAH-^H19^EMNVs or SAH-^H19^EMNVs together with RI (BMS-754807) at 7 and 14 days after operation. *p < .05 compared with control.

Microfil perfusion is a widely-accepted method to assess blood supply conditions (Guo et al., 2017, Tao et al., 2017c). Fourteen days after surgery, the formation of blood vessels in the untreated wounds and those treated with SAH, SAH-^293^EMNVs, SAH-^H19^EMNVs or SAH-^H19^EMNVs along with RI were examined by micro-CT. The reconstructed three-dimensional images (Figure 6(a)) showed a much higher density of blood vessels in the defects treated with SAH-^H19^EMNVs. Quantification of the blood vessels showed a significant increase in blood vessel area and blood vessel number in the defects treated with SAH-^H19^EMNVs compared to the untreated defects but the other groups did not show such significant therapeutic effects (Figure 6(b)).

Immunofluorescence (IF) staining of CD31 and α-SMA double staining, an acknowledged method to observe vascular reconstruction (Guo et al., 2017), revealed that the number of regenerated mature blood vessels in the dermal defect increased from post-operative day 7 to day 14 (Figure 6(c)). Quantification of the density of regenerated mature blood vessels also showed an increase from post-operative day 7 to day 14 in all five treatment groups (Figure 6(d)). The number of mature vessels in the defects treated with SAH-^H19^EMNVs was significantly higher than in the untreated defects at 14 days after operation but none of the other groups showed such significant therapeutic effects.

## Discussion

The results of the present research illustrate that the interdiction of LncRNA-H19 is critical for the hyperglycemia-induced impairment of angiogenesis by regulating the vitality of Akt activation, and the use of ^H19^EMNVs successfully sustained the angiogenic ability. Moreover, congruent results from both *in vitro* and *in vivo* experiments indicated that ^H19^EMNVs would be a potential specific treatment for diabetic wounds and other diabetic complications.

Various types of nanocarriers, ranging from 10 nm to 1 μm in diameter, have been manufactured, including polymeric particles and liposomes (Peer et al., 2007, Liu et al., 2016, Xing et al., 2016). In addition, the use of bioinspired or bioengineered delivery systems, including extracellular vesicles (EVs), has been proven to be an efficient and inexpensive delivery system for microRNA by virtue of the availability of overexpression technology (Tao et al., 2017a,d). For this reason, we chose overexpression technology and bioengineered delivery systems to inexpensively generate and deliver LncRNA-H19.

However, there is a serious drawback to the clinical use of EV-based medicine, which is the low-production rates of the EVs isolated from cells (Oh et al., 2015). In order to obtain a higher yield to meet clinical requirements, EMNVs, which are produced by serial extrusion (Hwang et al., 2015; Lunavat et al., 2016), became the first choice to deliver therapeutic LncRNA-H19.

Although there have been a large number of studies focused on packaging and delivery of siRNA (Guo et al., 2016; Lee et al., 2016; Yu et al., 2016), short hairpin RNA (Zhang et al., 2017) and microRNA (Yang et al., 2016), there are few reported studies focused on packaging and delivering of LncRNA. The results of the present pioneering study show that an EMNV-based delivery system can successfully package LncRNA-H19.

In glioma, LncRNA-H19 was upregulated in microvessels from glioma tissues and in glioma-associated endothelial cells (ECs) cultured in glioma-conditioned medium, while knockdown of LncRNA-H19 suppressed glioma-induced angiogenesis (Jia et al., 2016). In this study, ^H19^EMNVs rescued the hyperglycemia-induced reduction of LncRNA-H19 and proliferation *in vitro.* All these results implied the potential of LncRNA-H19 as a therapeutic target to treat diabetic wounds by promoting angiogenesis.

Wound healing is a physiological reparative response to injury and a well-orchestrated process that involves hemostasis, cell migration, proliferation, angiogenesis, extracellular matrix deposition, wound contraction and re-epithelialization (Icli et al., 2016). Favorable vascularization is necessary during the formation of new tissue, especially for chronic wounds (Kao et al., 2011). Blood vessels transport nutrients and oxygen to cells and tissues, remove metabolic waste, and supply soluble factors and circulating stem/progenitor cells, which are critical to tissue regeneration (Eming et al., 2007). As a result, appropriate vascularization is desirable and is particularly important for chronic wound healing. However, chronic wounds have a reduced response to the microenvironment during the healing process (Werdin et al., 2008), and this normally results in impaired angiogenesis (Mendez et al., 2015).

This reduction in angiogenesis could be attributed to hyperglycemia-induced impairment of Akt activation (Vind et al., 2012) and defects in angiogenesis(Larger et al., 2004) caused by H19-related impairment of the insulin-PI3K-Akt pathway (Gao et al., 2014). We next aimed to affirm that the interdiction of LncRNA-H19 is an important step related to hyperglycemia-induced impairment of angiogenesis, and a promising therapeutic target. Our experimental results confirm that LncRNA-H19 is indispensable for vascular regeneration; nevertheless, hyperglycemia diminishes the capacity for neovascularization by destabilizing the expression of LncRNA-H19. Fortunately, ^H19^EMNV therapy successfully stabilized the expression level of LncRNA-H19 and further ensured the normal function of vascular cells even in abnormally high blood sugar conditions.

Considering that, according to our observations, the diabetic-induced impaired angiogenesis was the primary cause of impaired full thickness wound healing, especially of the dermis – the deep vascular inner layer of the skin – rather than the epithelium, this situation could be described as ‘shiny on the “outside”, broken on the “inside”’. Consequently, it is important to evaluate not only wound closure speed, which is more intuitive, but also histological assessments of the structure of the regenerated skin, including HE staining, Masson’s trichrome staining and K14 staining.

In addition, H19 seems to perform additional roles in tissue repair. For example, H19 can activate TGF-β1 signaling and promote tendon regeneration (Lu et al., 2016). Interestingly, the activation of TGF-β1 signaling can also promote the expression of type I collagen (Lu et al., 2016) and participate in promoting the wound healing process (Guo et al., 2017). Hence, the supplementing of H19 via EMNVs is both an etiological treatment of the primary cause – impaired angiogenic ability – and a supportive treatment of other associated tissues.

In addition, abnormal inflammation is one of the ‘accomplices’ causing the impaired wound healing (Eming et al., 2014) via significant increases in IL-1β and TNFα (Tarnuzzer & Schultz, 1996). However, investigation of the underlying cause has found that the persistence of a high bacterial load in wounds is the ‘arch-criminal’ responsible for the abnormally increased inflammation and delayed healing (Frank et al., 2009; Roche et al., 2012; Eming et al., 2014). Further, it is widely known that an adequate blood supply is a necessary prerequisite for initiation of the immune response to pathogens and subsequent wound repair (Frantz et al., 2005), but the regeneration of the blood supply is impaired in diabetic wounds, as revealed in our study and in previous research (Marrotte et al., 2010; Xu et al., 2014). In conclusion, the wound healing process is like the music of a ‘symphony orchestra’ produced by the ‘concert performers’, including immune responses and ECM formation, and the ‘bandmaster’ of angiogenesis (Liu & Velazquez, 2008; Eming et al., 2014; Flegg et al., 2015). Hence, if H19 is so crucial to the process of angiogenesis, it is inevitable that it will impact diabetic wound inflammation.

Alginate has been used in numerous biomedical science and engineering applications because of its biocompatibility and ease of gelation (Sun et al., 2012), and the hydrogel form has been particularly attractive for use in drug delivery and wound healing (Lee & Mooney, 2012). There is even research indicating that sodium alginate hydrogel could be used to load exosomes (Guo et al., 2017). Because of these beneficial properties, sodium alginate was adopted in this study as a carrier to transfer EMNVs to the wound bed without causing any adverse reaction. In the present study, we confirmed that SAH is applicable for local administration of EMNVs.

According to our results, the membrane of EMNVs would protect nucleic acids from the complex constituents of body fluid including RNAses before they are taken up by cells. After being internalized by cells either by fusion with the cytoplasmic membrane or via an endocytic process just like EVs (Mulcahy et al., 2014), the cargos may be transferred functionally into the target cells in the same way as EVs (Abels & Breakefield, 2016).

There are still some mysteries associated with EMNVs such as, in particular, the generation mechanism. There are two possible mechanisms, interestingly, analogous to the two major types of EVs – exosomes (Exos) and micro-vesicles (MVs).

In the biogenesis of Exos, the ‘inward-budding’ of vesicles into the endosome forms multi-vesicular bodies (MVBs) (Maas et al., 2017). The vesicles in MVBs are named intraluminal vesicles (ILVs) (Maas et al., 2017), and when MVBs fuse with the cytomembrane, the released vesicles are named Exos (Maas et al., 2017). One classic mechanism of Exos biogenesis involves the endosomal sorting complex required for transport (ESCRT) machinery, and TSG101 (also named ESCRT-I) is essential for Exos formation (Schoneberg et al., 2017; Tao et al., 2017b). Tetraspanins including CD9, CD63, and CD81 are especially enriched on the membrane of Exos, because they participate in an ESCRT-independent mechanism of Exos biogenesis or cargo selection of Exos (Andreu and Yanez-Mo, 2014; Zhang et al., 2015). In conclusion, CD63, Tsg101, CD9 and CD81 are very specific markers for identifying Exos.

In the biogenesis of MVs, the ‘outward-budding’ vesicles are formed on the surface of the cell membrane (Maas et al., 2017). The cytomembrane, a lipid bilayer, is extruded and extended when cells pass through the pores of the filter, and the increasing tensile force ultimately causes the deformation and fragmentation of the cytomembrane; the fragments of lipid bilayer then self-assemble into hollow vesicles (Jo et al., 2014). In consideration of the origin of these self-assembled EMNVs, we named them ‘MV-like subtype of EMNVs’. Beyond that, we assume that ILVs, or intracellular Exos, will be released, without destruction, from MVBs after extrusion, because their size of 30–100 nm (Tao et al., 2017b) is much smaller than the pore diameter of the filter. Since they are derived from MVBs, we named them ‘Exo-like subtype of EMNVs’. The subtypes of EMNVs are shown schematically in Figure S2. In consideration of these potential subtypes of EMNVs, there is a need for further study to improve understanding and comprehension in the future.

## Conclusions

In this paper, we report the use of a potentially powerful tool, extracellular vesicle-mimetic nanovesicles (EMNVs) as a promising carrier of long noncoding RNA H19 (LncRNA-H19), in helping patients manage their diabetic wounds via the competing endogenous RNA (ceRNA) effect.

*In vitro*, the effect of ^H19^EMNVs on proliferation, migration and tube formation of ECs in high glucose conditions was examined by flow cytometry with an EdU kit, CCK-8 assay, transwell assay and tube formation assay. The results showed that ^H19^EMNVs rescued EC proliferation, migration and tube formation which were impaired by high glucose. After more in-depth research, we confirmed that the interdiction of LncRNA-H19 is the ‘pivotal point’, which is induced by high glucose and could cause the impairment of angiogenesis via impairment of Akt activation. Thus, ^H19^EMNVs constitute a powerful precision-medicine therapeutic tool aimed to ameliorate the causes of diabetic chronic wounds.

Alginate hydrogel-loaded ^H19^EMNVs were applied as a dressing on the wound bed of an *in vivo* experimental model to observe whether it had the ability to accelerate angiogenesis and wound healing *in vivo*. The result showed that ^H19^EMNVs obviously improved the blood supply of chronic wounds, promoted re-epithelization and helped the process of wound healing.

In conclusion, these findings highlight both the role of EMNV as a high-yield bioengineered nano-drug delivery system for biomacromolecules and the effect of LncRNA as an epigenetic regulatory system via the ceRNA network in promoting regeneration and repair of wounded tissue.

## Supplementary Material

IDRD_Guo_et_al_Supplemental_Content.docx
