# Supplementary material for: Extracellular vesicle-mimetic nanovesicles transport LncRNA-H19 as competing endogenous RNA for the treatment of diabetic wounds
Source: Drug Deliv. 2018 Jan 15;25(1):241–55. doi: 10.1080/10717544.2018.1425774 (PMC6058500; doi:10.1080/10717544.2018.1425774)
Supplement: IDRD_Guo_et_al_Supplemental_Content.docx [file IDRD_A_1425774_SM2901.docx]

**Supplemental materials**


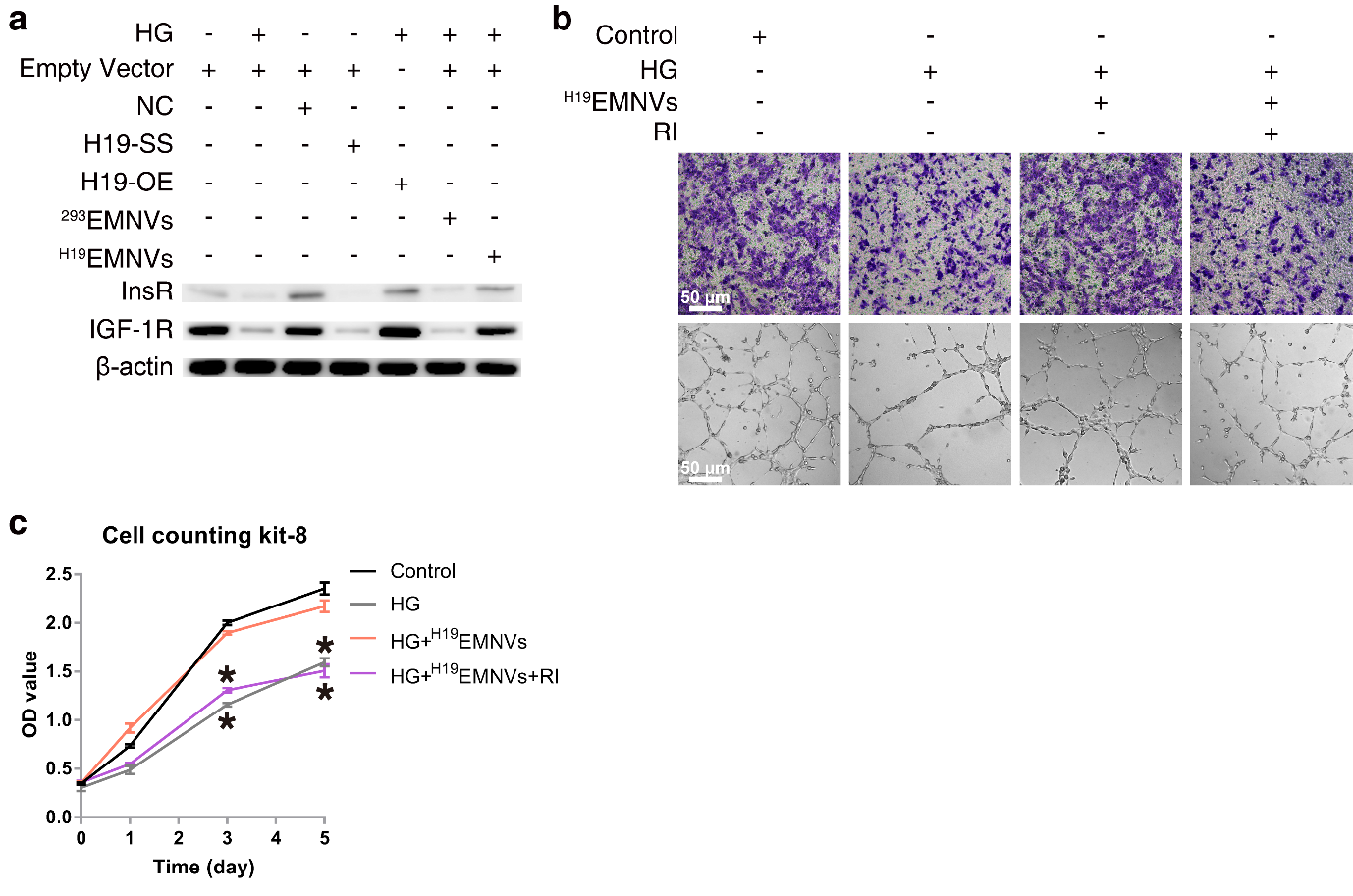


**Figure S1**. (**a**) Western blot analysis of the expression level of InsR and IGF-1R. (**b**) Representative photomicrographs of transwell assays; scale bar, 50 μm, and representative photomicrographs of tubule formation; scale bar, 50 μm. (**c**) Proliferation of HMEC-1 detected using a CCK-8 kit on day 0, 1, 3 and 5. **P* < 0.05 compared with control.


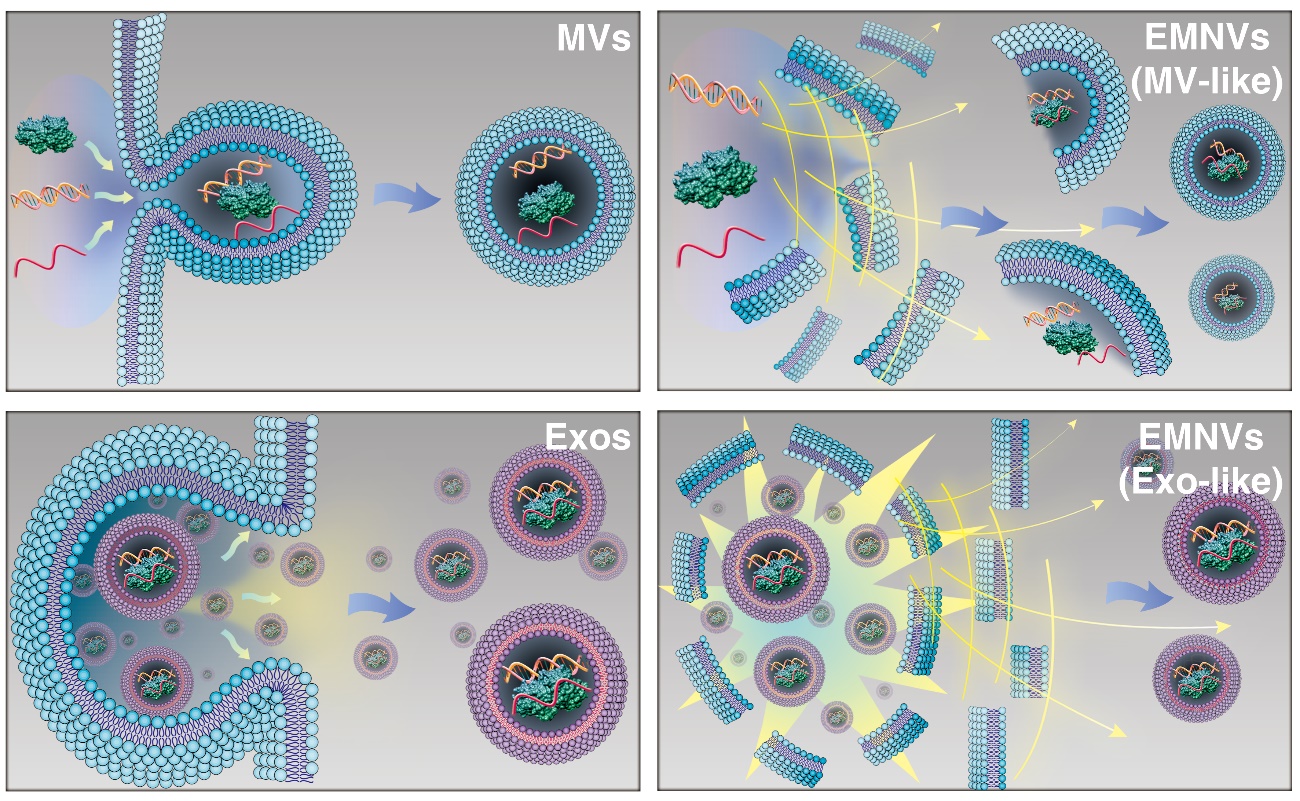


**Figure S2. Schematic diagram of the possible formation mechanism of EMNVs.**
